# Implementation of a flow protocol in home care for children with special healthcare needs

**DOI:** 10.1590/0034-7167-2025-0003

**Published:** 2025-11-03

**Authors:** Mariana Matias Santos, Beatriz Rosana Gonçalves de Oliveira Toso, Altamira Pereira da Silva Reichert, Paloma Mayara Vieira de Macena Lima, Eliane Tatsch Neves, Regina Aparecida Garcia de Lima, Elenice Maria Cecchetti Vaz, Neusa Collet

**Affiliations:** I Universidade Federal de Pernambuco. Recife, Pernambuco, Brazil; II Universidade Estadual do Oeste do Paraná. Cascavel, Paraná, Brazil; III Universidade Federal da Paraíba. João Pessoa, Paraíba, Brazil; IV Universidade Federal de Santa Maria. Santa Maria, Rio Grande do Sul, Brazil; V Universidade de São Paulo. Ribeirão Preto, São Paulo, Brazil

**Keywords:** Home Care Services, Workflow, Child Health, Chronic Disease, Nursing., Servicios de Atención de Salud a Domicilio, Flujo de Trabajo, Salud Infantil, Enfermedad Crónica, Enfermería.

## Abstract

**Objectives:**

to validate and implement a home care flow protocol for children with special healthcare needs.

**Methods:**

mixed-method research conducted in home care services in Paraíba, Brazil, with 13 judges for content validity and 11 interviews with healthcare professionals after clinical implementation. Quantitative data were analyzed by calculating the Content Validity Index and Cronbach’s alpha, and qualitative data were analyzed by inductive thematic analysis. Mixed-method analysis was performed by integrating data in a joint display.

**Results:**

the instrument was considered substantial and valid, with a Cronbach’s alpha of 0.87. There was a need for adjustments to the protocol flowchart in the “transportation”, “communication with Primary Health Care”, “Singular Therapeutic Project implementation”, “telecare” and “emergency service activation” items.

**Conclusions:**

the protocol was considered functional for use by home services after incorporation of the suggested adjustments.

## RESUMEN

**Objetivos:**

validar e implementar un protocolo de flujo de atención domiciliaria para niños con necesidades especiales de salud.

**Métodos:**

investigación de métodos mixtos realizada en servicios de atención domiciliaria en Paraíba, Brasil, con 13 jueces para la validación de contenido y 11 entrevistas con profesionales de la salud tras la implementación clínica. Los datos cuantitativos se analizaron mediante el cálculo del Índice de Validación de Contenido y el alfa de Cronbach, y los datos cualitativos se analizaron mediante análisis temático inductivo. El análisis de métodos mixtos se realizó integrando los datos en una visualización conjunta.

**Resultados:**

el instrumento se consideró sustancial y válido, con un alfa de Cronbach de 0,87. Se observó la necesidad de realizar ajustes en el diagrama de flujo del protocolo en los ítems “transporte”, “comunicación con Atención Primaria”, “implementación del Proyecto Terapéutico Singular”, “teleasistencia” y “activación del servicio de urgencias”.

**Conclusiones:**

tras la incorporación de los ajustes sugeridos, el protocolo se consideró funcional para su uso en servicios domiciliarios.

## INTRODUCTION

Advances in science and healthcare have influenced changes in the profile of child morbidity and mortality worldwide. This is particularly true in developing countries, with a significant increase in the survival of children with special healthcare needs (CSHCN), as well as improved control and management of acute diseases^(1)^.

International studies highlight that although CSHCN represent a minority of the population, they are admitted more frequently by emergency services and experience more hospital admissions than the general pediatric population^(2,3)^. However, care for CSHCN does not always need to be carried out in hospital units, where children can be exposed to various types of infections and trauma, increasing healthcare costs, sometimes unnecessarily^(4)^. Research carried out in the United States of America indicates that multiple hospitalizations considerably reduce the survival of CSHCN^(5)^.

Therefore, care for CSHCN cannot be restricted to hospital environments, with a need to extend it to home environments as well. The transition process from hospital to home requires support and provision of family-centered care and home care (HC)^(3)^. This is an important strategy for providing care for CSHCN, as it enables the provision of care in their social and family environment, and longitudinal support to caregivers. In Brazil, it has three modalities: HC1, under the responsibility of primary care; and HC2 and HC3, under the responsibility of home care service (HCS)^(6)^.

A study shows that HC, in addition to promoting dehospitalizations, also centralizes care in the family, offering support for developing skills with necessary guidance to train primary caregivers, including information about the signs and symptoms of disease exacerbation. Furthermore, it considers home environments as an important element for children’s recovery, being able to provide expanded, humanized and comprehensive care through the establishment of a Singular Therapeutic Project (In Portuguese, *Projeto Terapêutico Singular* - PTS)^(7)^, in addition to contributing to the promotion of healthy child development.

In Brazil, the structuring of this service is relatively recent, from 2013, therefore lacking organization in the transit and flow from hospital to home of patients in general and CSHCN in particular. A pilot study developed in the state of Paraná, Brazil, developed a proposed flowchart for care for CSHCN in HCS^(8)^. Seeking to expand knowledge about this population in other national scenarios, the validity of the aforementioned protocol in other states, such as Paraíba, is a strategy that aims to expand its use.

Considering the above, the following guiding question arose: is the CSHCN care flow protocol adequate for organizing care flow in HCSs in the state of Paraíba?

## OBJECTIVES

To validate and implement a home care flow protocol for CSHCN.

## METHODS

### Ethical aspects

The research was certified by the Research Ethics Committee. All ethical precepts of Resolution 466/2012 were respected. To ensure anonymity, participants were identified with the initial letter corresponding to their professional category (SW - social worker; N - nurse; PH - physiotherapist; ST - speech therapist; P - physician; NU - nutritionist; PS - psychologist), followed by the number according to the sequence in which the study was conducted.

### Study design, period and place

This is an explanatory sequential mixed method study QUAN→qual, developed in two dependent phases^(9)^. The research followed the recommendations of a checklist for mixed methods^(10)^. The quantitative stage was necessary to perform flow protocol content validity for HCS for CSHCN. The subsequent qualitative stage explored the trends in the use of a flow protocol as well as the interpretation of evidence from quantitative data.

Data collection took place in the authorized HCSs in the state of Paraíba until July 2019, which met CSHCN’s spontaneous demand, comprising a total of nine municipalities. Among these municipalities, 13 teams included the two modalities provided: multidisciplinary home care team (MHCT); and multidisciplinary support team (MSPT).

### Population or sample; inclusion and exclusion criteria

One representative per team participated as a judge in the quantitative stage, totaling 13 professionals, who had an academic degree in one of the professional categories that make up HCS, with or without specialization, a master’s and/or a doctoral degree, and a minimum of two years of professional experience in assistance in HCS. Those who were on leave and/or vacation during the data collection period were excluded.

In the qualitative stage, training workshops were held for professionals to develop the clinical implementation of the protocol in a pilot HCS in the state of Paraíba, Brazil, in March and April 2021. The workshops took place after prior scheduling with 32 higher education professionals who were part of MHCT or MSPT and who had already assisted or were assisting CSHCN. Professionals who did not have time availability compatible with the researcher and/or who were on vacation or away from work were excluded from the workshops.

### Study protocol

The study began by sending an invitation to participate to the 13 HCS professionals by email as well as the standardized instrument for validity via Google Forms^®^. In this instrument, the judge reported personal characteristics (sex, age), academic background (time since graduation, length of experience, specializations) and assessment regarding aspects related to relevance, clarity, objectivity, simplicity and accuracy of 31 items assessed.

After content validity analysis, adjustments were made to item 3, in which the terms “HC2” and “HC3” were inserted; in item 5, in which the possibility of referral by nurses, physicians, physiotherapists, social workers, nutritionists and psychologists from the hospital and/or PHC was included; and in item 31, in which the terms “HC2” and “HC3” were inserted. Then, the qualitative stage began.

At this stage, six workshops were held with an average duration of 60 minutes per meeting. All were guided by a previously structured intervention plan, based on David Ausubel’s “meaningful learning” theoretical framework^(11)^. Two months after the clinical implementation of the flowchart, in October 2021, professionals who had participated in the workshops and who had had experience in caring for CSHCN in the months following the workshops were invited to participate in the interviews, making up a total of 11 healthcare professionals, meeting the sufficiency criterion^(12)^.

The interviews were conducted by the lead researcher, who has experience in research and who participated in HCS pilot, by prior appointment and in a private environment. The script used was divided into two chunks: the first contains data regarding professional sociodemographic characteristics; and the second contains the following guiding questions: how do you assess the functioning of the flow protocol for caring for CSHCN by HCS? What is your perception about the possibility of implementing the flow protocol for caring for CSHCN by HCS? Would you change anything in the flow protocol so that it better meets your reality? If so, what and why?

The audios were recorded on digital media and lasted an average of 1 hour and 10 minutes. After being transcribed in full, the interviews were given to participants so that they could analyze the content, and they could add or refute information.

### Analysis of results, and statistics

**Figure 1:**
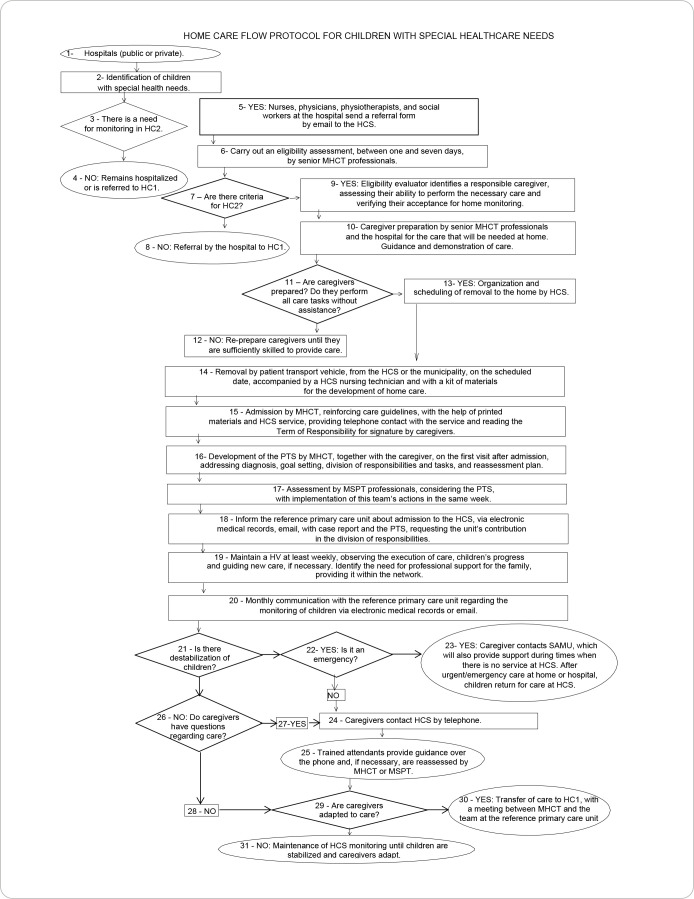
Proposed flowchart for caring for children with special healthcare needs in home care, containing 31 numbered and assessed items, João Pessoa, Paraíba, Brazil, 2022

*Source: Rossetto, Toso and Rodrigues (2020)^(8)^.*

In quantitative analysis, agreement with instrument items was assessed by the Content Validity Index (CVI), with a minimum threshold for item acceptance of 80%. For instrument construct validity, the reliability measure of judges’ opinions was used, and reliability was expressed by Cronbach’s alpha, whose value was 0.87, demonstrating that the flowchart has good reliability according to judges’ judgment. Calculations were performed using the statistical software R^(13)^ of the Statistical Package for the Social Sciences version 20.0. Each item assessed will be demonstrated in the flowchart by means of Figure 1.

Qualitative data were subjected to inductive thematic analysis, which is developed in six stages^(14)^. Initially, the interview data was familiarized through transcription and successive readings, recording first impressions. Subsequently, the data were extracted and coded. These codes were grouped according to their affinity to generate pre-topics and topics. At the end of this phase, the material was reviewed to identify gaps in the process of structuring the interview excerpts. Thus, it was possible to identify intra-topic and inter-topic gaps to refine them so that they presented a coherent and cohesive pattern among themselves and with the other topics constructed. Then, the following topics were constructed: *implementation of care flow protocol for CSHCN: items to be improved;* and *challenges for HCS in managing care for CSHCN according to the flow protocol.* Furthermore, all audio transcripts of interviews, as well as the assessment of topics, were consensually verified for accuracy by at least two authors.

After analyzing quantitative and qualitative data separately, meta-inferences were made by mixing and comparing the CVI values, the items assessed by judges, and the extracts from the speeches of the professionals interviewed. The data from the analysis of convergent and divergent items were presented in joint displays.

## RESULTS

Quantitative and qualitative data integration allowed identifying convergent and divergent points between content validity and analysis of professionals’ perception. Thus, data convergence was identified in item 5 of the protocol, with a median CVI and statements that refer to the non-routine use of referral forms by institutions to HCS via email. The forms for activating HCS are made available via email or delivered in person by both the hospital and Primary Health Care (PHC), and sometimes, telephone contacts are made, requiring adjustment of an item.

In item 11, regarding caregiver preparation, the need for supplementation with the inclusion of analysis of home environment and structure as an important condition for hospital discharge stood out. Moreover, concern was identified in visiting the home to assess the conditions of an environment to receive CSHCN and the establishment of agreements with the care network in view of the needs of the admitted children.

Item 14, which addresses hospital removal using a HCS transport vehicle accompanied by a nursing technician, was not validated in both stages of the research. It was found to be impossible to implement it due to the lack of the service’s own transport properly equipped for this purpose and the lack of safety of a technician when carrying out removal.

Item 20, which guides monthly communication with PHC, was assessed as inadequate in both stages of the research. Qualitative extracts broaden the possibility of interpretation by highlighting disarticulation between HCS and PHC, with non-routine communication among teams and lack of interest by the PHC team.

Finally, in item 29, which provides guidance on caregiver adaptation as a criterion for discharge from HCS, there was also convergence in both stages, given that this was considered a sufficient criterion for safe discharge. Chart 1 presents the aforementioned data, which contains joint display with convergent data.

**Chart 1:** Joint display comparing convergences between Content Validity Index and narratives in in-depth interviews with home care service professionals, João Pessoa, Paraíba, Brazil, 2021

Items	CVI	Interviews’ narratives
Item 5 - YES: Nurses, physicians, physiotherapists, and social workers at the hospital send a referral form by email to the HCS.	61.5	*Many times, we don’t receive the form, we actually receive the request. They come to speed up the process because, many times, it is sent by email and takes a long time to be printed. So, they call and we schedule an assessment.* (N3)
Item 11 - Are caregivers prepared? Do they perform all care tasks without assistance?	76.9	*Often, specific equipment is needed for the child to be able to stay at home. We analyze this real need, to find out if they are able to be monitored by HCS at home.* (NU10) *The MHCT first goes to the hospital to inventory the equipment and, if it needs any that we do not have, we contact the appropriate agencies so that they actually release the equipment and the child can go home more safely.* (PS2)
Item 14 - Removal by patient transport vehicle, from the HCS or the municipality, on the scheduled date, accompanied by a HCS nursing technician and with a kit of materials for the development of home care.	76.9	*There is the nursing council issue* [...]. *A while ago, we had some difficulties. The nursing technicians here did not feel safe if they were not with the nurse to remove the patient, because our patients are complex, they are not simple patients. The transfer of a patient from the hospital environment, who is stable, but who at any moment can get complicated.* (PH4) *If HCS does not have transportation, we have no way of doing it* [...]. *From the point of view of the flow* [flow protocol], *it is very good, but we need to adapt the ideal to the possible.* (P6)
Item 20 - Monthly communication with the reference primary care unit regarding the monitoring of children via electronic medical records or email.	76.9	*I don’t have monthly* [communication with USF]. *It would be good if it were, but the “rush” doesn’t allow it. There are many patients with many demands, because we don’t only see children.* (N9) *There’s no point in having this initiative* [periodic communication]. *There have been many cases where, in practice, we have this very good relationship and we approach* [the USF] *and, in fact, we are not well received. If we were to hold, for instance, monthly meetings, I’m absolutely sure they wouldn’t even look at it, because we already bring the medical records; people don’t even look at the paper.* (PH5)
Item 29: Are caregivers adapted to care?	92.3	*If this child is stabilized, the family is well-guided, and follows care appropriately, we know that they will continue with what was advised, and that they have the support of the family health unit. That this service* [PHC] *can continue.* (NU10)

*CVI - Content Validity Index.*

In item 6, which addresses the assessment of eligibility for admission to HCS, discrepancies were identified in the comparison between the quantitative and qualitative stages, since, despite the validity of judges, the qualitative stage revealed that the time of up to one week to carry out this assessment is not met.

A discrepancy was also identified in item 10, because although joint preparation between the hospital and HCS team for the discharge of CSHCN is recommended and established in the protocol, this did not happen in care practice. The unavailability of MHCT professionals and the hospital to prepare caregivers jointly was evidenced, with hospital professionals being responsible for this preparation before discharge.

Concerning guidance for developing the PTS by the MHCT together with caregivers, in the first home visit (HV) after admission, as suggested by item 16 of the flowchart, previously validated by judges, divergences were also identified related to the PTS construction, i.e., the frequency of implementation, the one-week time for the first PTS discussion and the family’s participation in construction.

Item 18 addresses communication about admission to PHC, via medical records or email with sharing of the PTS, validated in the quantitative stage, but divergent in the qualitative stage due to lack of regular communication with PHC, impossibility of using formal electronic communication channels, and difficulty in sharing responsibility between HCS and PHC.

In relation to the frequency of HV and care for caregivers, item 29 was validated by judges, but not implemented in care practice. The divergences found were related to the frequency of HV and support for caregivers to meet their health demands, as there is a lack of coordination to guarantee access to healthcare services.

In item 23, regarding Mobile Emergency Care Service (In Portuguese, *Serviço de Atendimento Móvel de Urgência* - SAMU) activation, difficulties or even refusal to provide care in cases of child destabilization were noted. SAMU should be the support of HCS during times when the service is not available.

Obstacles were identified for the provision of telecare by a trained attendant in item 25 of the flow protocol. Participants claimed a lack of professionals exclusively for this activity, indicating the need to hire professionals for the proper implementation of telecare. Chart 2 displays these discrepancies.

**Chart 2:** Joint display comparing divergences between Content Validity Index and narratives in in-depth interviews with home care service professionals, João Pessoa, Paraíba, Brazil, 2021

Items	CVI	Interviews’ narratives
Item 6 - Carry out an eligibility assessment, between one and seven days, by senior MHCT professionals.	100	*We try to do it as quickly as possible* [eligibility assessment]. *We don’t do much in up to a week. It depends on whether we have a lot of patients, who already have a* [previously] *scheduled appointment.* (P6)
Item 10 - Caregiver preparation by senior MHCT professionals and the hospital for the care that will be needed at home. Guidance and demonstration of care.	100	*The hospital team trains* [in the hospital environment for discharge]. *We finish training at home, because we cannot intervene in the conduct of hospital professionals. So, we do this training when we are at home* [with the child]. *But the hospital team is the one who trains the caregiver, the patient still being hospitalized.* (PH4)
Item 16 - Development of the PTS by MHCT, together with the caregiver, on the first visit after admission, addressing diagnosis, goal setting, division of responsibilities and tasks, and reassessment plan.	100	*I still haven’t managed to do it together with caregivers* [to build the PTS]. *This proposal* [of the first visit within one week from activation] *cannot be achieved. If we extend the time a little longer, we can include two professionals to make this visit, but we can’t do it in seven days. Two weeks is enough.* (N3)
Item 18 - Inform the reference primary care unit about admission to the HCS, via electronic medical records, email, with case report and the PTS, requesting the unit’s contribution in the division of responsibilities.	92.3	*This is not done regularly* [communication with PHC]. *But what we feel we need to share* [depending on specific needs identified]*, we immediately schedule, as well as when we need to close our stay there* [when the demand is resolved]. *So, generally, we make contact.* (P6)
Item 19 - Maintain a HV at least weekly, observing the execution of care, children’s progress and guiding new care, if necessary. Identify the need for professional support for the family, providing it within the network.	100	*Usually, it is weekly* [frequency of HCS visits], *but it will depend on the clinical condition.* (N7) *We do not seek a professional for the caregiver. Sometimes, I recommend medication; if necessary, an antidepressant. I already tell her that she needs a psychologist, who we have on our team and can provide support to her. Now, if it is another type of professional, we encourage her to go to a FHS* [Family Health Strategy] *physician for them to see her.* (P11)
Item 23 - YES: Caregiver contacts SAMU, which will also provide support during times when there is no service at HCS. After urgent/emergency care at home or hospital, children return for care at HCS.	100	*The patient says, “Ah, I called SAMU, but SAMU didn’t come, SAMU said that wasn’t their job”. It’s as if the emergency service always disbelieves that the patient really needs the service. The history of many false phone calls created a barrier for professionals to see that call as something really necessary.* (P6)
Item 25 - Trained attendants provide guidance over the phone and, if necessary, are reassessed by MHCT or MSPT.	84.6	*The person who provides care is not qualified to guide the caregiver. The family contacts reception. Reception answers the call, takes notes and passes the situation on to us* [healthcare professionals], *and we contact them according to demand.* (NU10)

*CVI - Content Validity Index.*

## DISCUSSION

The mixing of quantitative and qualitative data made it possible to understand the adjustments needed to adapt the CSHCN service flow protocol to practical reality as well as to identify the obstacles that interfere with its functionality.

The first elements requiring adjustment in the protocol assessed concern activation and time for assessing CSHCN eligibility. The “Better at Home” program allows and encourages HCS activation by hospital-level services, by PHC, by emergency care units, specialty units and outpatient referrals, or even by the initiative and need of patients, family members and neighbors. It is also recommended that there be an organization of HCSs for assessing eligibility, with established deadlines^(6)^.

The protocol proposes a period of up to seven days to carry out this assessment. However, the need for adjustments in the teams’ schedules to meet the requests of the hospital and PHC generated the need to adjust eligibility assessment time to up to 15 days after HCS is activated.

After eligibility assessment, it is necessary to identify a caregiver responsible for CSHCN. The protocol proposes that caregivers be prepared jointly by the hospital and HCS teams, modifying the predominantly biomedical model of hospital discharge, enabling discharge to be organized together with the multidisciplinary team. An international study points to transitions from hospital-home-HC care as a possible cause of readmissions in children with chronic conditions, suggesting improvements in communication as a strategy for continuity of care^(15)^.

In the Brazilian reality, research has found that CSHCN dehospitalization can be influenced by the lack of planning according to children’s/families’ reality, as well as by unplanned discharges that generate problems in communication among teams, the health network and the family. It also suggests the implementation of institutional protocols as a potential way to improve dehospitalizations^(16)^. The preparation of caregivers for the development of HC by HCS and hospital professionals includes interprofessionalism for safe continuity of care, allowing discharge planning with HCS organization to monitor discharge from the hospital and provide HC^(17)^. One strategy for implementing this item could be the use of videoconferences in the days prior to hospital discharge; this would bring benefits such as sharing, transparency and humanization of care^(18)^.

Another element proposed in the protocol, related to the discharge schedule for CSHCN, is the performance of transportation after discharge by HCS ambulance or municipal health transport, accompanied by a nursing technician. The analysis of assessment of this item allowed us to identify convergence in the comparison between content validity and clinical implementation. In both, item inadequacy for the studied reality was evidenced, due to the lack of an ambulance exclusively for HCS, insecurity and doubts about the Federal Nursing Council (In Portuguese, *Conselho Federal de Enfermagem* - COFEN) regulations for the performance of this type of transportation accompanied by a nursing technician.

COFEN, in Resolution 588/2018, authorizes nursing technicians to transport patients in minimum or intermediate care and assigns a nurse the responsibility of assessing patients’ general condition, providing the necessary equipment for assistance during transport, defining the nursing professionals who will assist patients during transfer^(19)^ and carrying out continuing education as a strategy to enable the implementation of an item^(20)^.

As for the acquisition of equipped transport, feasibility requires coordination between HCS and municipal management, with municipality planning and financial organization, based on the inclusion of vehicle purchase in the Annual Budget Law followed by the implementation of a bidding process. Moreover, it is necessary to provide material resources and train HCS team to systematize the use of transport for transfers from hospital to home and to carry out other elective transfers whenever necessary. A literature review study demonstrated the need to reorganize the Brazilian Health System (In Portuguese, *Sistema Único de Saúde* - SUS) financial resources, due to the underfunding of public policies, which have harmed the right to health and comprehensive care^(21)^. The importance of budgetary reorganization is highlighted, with the purpose of obtaining specific vehicles for HCS that enable the safe transportation of patients accompanied by primary care, considering that health transport services and mobile emergency services are already responsible for supporting the tertiary and outpatient service in removals for carrying out exams and other procedures, as well as transfers.

In addition to removal upon discharge, CSHCN treated by HCS also occasionally require transportation to undergo examinations and/or complementary therapies. The ministerial ordinance that redefines HC guides, as a priority for municipal health managers, the flow of user transportation and removal in both elective and emergency situations^(22)^. Furthermore, Ordinance MoH 2,563/2017 guarantees financing for the use of elective health transport for SUS users^(23)^. The reality of its operation is permeated, however, by serious difficulties related to the use of transport for dehospitalization and removal of CSHCN, both in elective and emergency situations.

Gaps in communication among Health Care Network (In Portuguese, *Rede de Atenção à Saúde* - RAS) services were also identified, as neither the health units nor HCSs have implemented electronic medical records, which considerably hinders network communication. However, this reality is in the process of changing, due to the beginning of the implementation of the Digital Health Policy by the Ministry of Health (MoH), which prioritizes the computerization of the three levels of care by 2028^(24)^, considering that the communicative capacity provided by the use of electronic medical records facilitates communication and exchange of information among health teams^(25)^.

Furthermore, a Brazilian study identified as challenges for communication^(26)^ the lack of qualification of PHC professionals for care coordination, the lack of understanding about the eligibility criteria by HCS associated with the absence of guidance for maintaining communication among RAS points as well as the disproportionality between the service capacity and the population that needs to be assisted.

Research conducted with PHC professionals highlights the organization of spaces for formal meetings among teams as a strategy to overcome this communication obstacle as well as the need for public policies that guarantee mechanisms for collaborative work in PHC^(27)^. Communication is a strategic and essential element for comprehensive care by the multidisciplinary team, but it depends on professional ties and communication spaces between professionals and managers^(28)^.

From this perspective, the digital environment is a space that can be used to improve communication between PHC and HCS professionals by creating a formal communication platform through messaging applications, in which there would be an exchange of information about patients’ healthcare needs, facilitating continuity of care and communication among teams. To this end, despite the current investments by the MoH in information and communication technologies, government strategies and policies are still needed to expand access, training and coordination among healthcare professionals in their respective public services^(29)^.

In relation to PTS development in the first visit, it was possible to identify the lack of systematic and routine implementation, in addition to it not being considered for communication with PHC. The use of this tool guides the approach to the complexity of care, considering the clinical, family, spiritual and social issues that are involved in the conditions that alter health, also allowing the survey of CSHCN’s and families’ demands, directing them in the RAS, in addition to guiding the organization and frequency of HVs, facilitating exchange of knowledge, decision-making and uniqueness of the care provided^(26)^.

Regarding the frequency of visits, variability in routine was identified, depending on healthcare professionals’ assessment of CSHCN’s needs. A study carried out with family members of CSHCN treated by HCS indicated that the flexibility of weekly care is beneficial, as it allows for continuity of HC by adapting to the family routine^(30)^.

It is worth noting that the protocol does not propose to tighten care actions, and it is understandable that the maximum number of visits that can be carried out by teams to meet CSHCN’s demands should be adjusted. However, it is necessary to standardize the minimum period for carrying out visits of at least one visit per week, in addition to transferring patients who need less than four visits per month to HC1^(6)^.

From this perspective, there is a need to adapt the teams’ work process to meet the minimum target proposed by the MoH. The proposed protocol is a facilitating tool in this process, with frequent assessment of the situational reality, transferring to PHC CSHCN who no longer require care in the HC2 and HC3 modalities. The proposed instrument can be made more flexible based on the analysis of exceptions and specificities of care, having as an interventional basis the PTS constructed in a shared manner between the teams and the family.

Another point of divergence relates to situations in which the disease worsens in CSHCN monitored by HCS. Although the ministerial order guarantees the provision of transportation and access to emergency services, significant obstacles were observed in activating SAMU, with a lack of credibility among family members when they need an ambulance.

It is worth noting that SAMU professionals also face difficulties in their daily work, associated with the population’s lack of knowledge regarding SAMU’s function, gaps in the regulatory process and, sometimes, inadequacy of available resources with poor distribution of ambulances. Solving this problem requires strategies that improve communication between SAMU professionals and other RAS professionals^(31)^.

The provision of telecare by HCS may constitute a strategy that can directly influence the appropriate or inappropriate activation of SAMU. Telecare is addressed in the flow protocol as a strategy for clarifying doubts, without an emergency nature^(8)^. However, obstacles to telecare implementation were identified due to the lack of trained attendants available to perform this function and the absence of a mobile phone for this purpose. A systematic review study demonstrates the use of the remote support strategy for patients with chronic conditions associated with a better quality of life^(32)^.

Concerning the discharge of CSHCN from HCS, it was identified that caregivers’ adaptation to continue care at home was considered a valid and sufficient criterion for referring CSHCN to HC1. However, the lack of trust and the absence of a bond between CSHCN, their families and PHC are identified as conditions that cause the transfer of responsibility from PHC to secondary care services, due to the lack of preparation and insecurity of healthcare professionals and the disarticulation of the RAS, impairing the longitudinality of care^(33)^.

Reflecting on the clinical implementation of the proposed flow protocol highlights difficulties in daily practice. However, its use aims to guarantee equal rights within the RAS to the entire population of assisted CSHCN.

### Study limitations

The limitations of this study include the implementation in only one municipality in the state, due to the difficulties faced in carrying out the research during the pandemic period (COVID-19). It is important to assess the flowchart in other municipalities and states for possible adaptation for use in the national territory with the appropriate articulations and agreements with the other points of the RAS.

### Contributions to nursing, health or public policy

The validity of the flow protocol for CSHCN care helped to understand the complexity of care for CSHCN assisted by HCS. The organizational protocol will enhance health resources, empowering professionals and users, in addition to providing support for CSHCN flow management in HCS, contributing to the longitudinality of the care offered to this population.

## CONCLUSIONS

The adequacy of the proposed flow protocol is subject to organizational adjustments, such as including caregivers in the assessment of CSHCN’s needs, increasing the number of days for assessing eligibility, and adjusting the schedules for the frequency of visits and team meetings. Moreover, adjustments are needed in the coordination between HCS, hospital and PHC, as well as the provision of materials and resources for the proper functioning of HCS.

Adjustments to the flow protocol to ensure the functionality of the proposed health technology are a complex demand, but feasible and useful for the setting studied. However, it is worth highlighting that the structural and communication barriers identified generate overload of equipment and healthcare services, as well as compromise the continuity of care for CSHCN between the different points of the RAS. After comparative data analysis and understanding the factors that influence immediate practical applicability, the care flow protocol for CSHCN was assessed as functional for use by HCS in the care of these children. This care is a dynamic and interactive process that requires coordination, empathy and quality public management.

## Data Availability

The research data are available within the article.
