# Supplementary material for: Implementation of a flow protocol in home care for children with special healthcare needs
Source: Rev Bras Enferm. 2025 Nov 3;78(Suppl 2):e20250003. doi: 10.1590/0034-7167-2025-0003 (PMC12584954; doi:10.1590/0034-7167-2025-0003)
Supplement: Translation

# Translation: Implementação de protocolo de fluxo na atenção domiciliar para crianças com necessidades de saúde especiais

**DOI:** 10.1590/0034-7167-2025-0003pt

**Authors:** Mariana Matias Santos, Beatriz Rosana Gonçalves de Oliveira Toso, Altamira Pereira da Silva Reichert, Paloma Mayara Vieira de Macena Lima, Eliane Tatsch Neves, Regina Aparecida Garcia de Lima, Elenice Maria Cecchetti Vaz, Neusa Collet

**Affiliations:** I Universidade Federal de Pernambuco. Recife, Pernambuco, Brasil; II Universidade Estadual do Oeste do Paraná. Cascavel, Paraná, Brasil; III Universidade Federal da Paraíba. João Pessoa, Paraíba, Brasil; IV Universidade Federal de Santa Maria. Santa Maria, Rio Grande do Sul, Brasil; V Universidade de São Paulo. Ribeirão Preto, São Paulo, Brasil

**Keywords:** Serviços de Assistência Domiciliar, Fluxo de Trabalho, Saúde da Criança, Doença Crônica, Enfermagem.

## Abstract

**Objetivos:**

validar e implementar protocolo de fluxo de atenção domiciliar às crianças com necessidades de saúde especiais.

**Métodos:**

pesquisa de método misto, realizada nos serviços de atenção domiciliar da Paraíba, Brasil, com 13 juízes, para validação de conteúdo, e 11 entrevistas com profissionais de saúde, após implementação clínica. A análise dos dados quantitativos foi realizada por meio do cálculo do Índice de Validação de Conteúdo e alfa de Cronbach, e dos dados qualitativos, por análise temática indutiva. A análise mista ocorreu por integração dos dados em *joint display*.

**Resultados:**

o instrumento foi considerado substancial e válido, com alfa de Cronbach de 0,87. Houve necessidade de adequações no fluxograma do protocolo nos itens “transporte”, “comunicação com a Atenção Primária à Saúde”, “realização do Projeto Terapêutico Singular”, “teleatendimento” e “acionamento do serviço de urgência”.

**Conclusões:**

o protocolo foi considerado funcional para utilização pelos serviços domiciliares após incorporação dos ajustes sugeridos.

## INTRODUÇÃO

O avanço da ciência e das formas de cuidados em saúde tem influenciado a modificação do perfil de morbimortalidade infantil em todo o mundo. Isso é factível, sobretudo, nos países em desenvolvimento, com significativo aumento da sobrevida de crianças com necessidades de saúde especiais (CRIANES), além de melhor controle e enfrentamento das enfermidades agudas^(1)^.

Estudos internacionais ressaltam que, apesar de as CRIANES representarem minoria da população, elas são admitidas com maior frequência pelos serviços de urgência e emergência, e vivenciam mais internações hospitalares que a população pediátrica em geral^(2,3)^. Todavia, nem sempre os cuidados às CRIANES precisam ser realizados em unidades hospitalares, nas quais as crianças podem ser expostas a diversos tipos de infecções e traumas, elevando os custos de saúde, algumas vezes de forma desnecessária^(4)^. Pesquisa realizada nos Estados Unidos da América aponta que múltiplas internações diminuem consideravelmente a sobrevida de CRIANES^(5)^.

Diante disso, o cuidado de CRIANES não pode se restringir ao ambiente hospitalar, havendo a necessidade de ampliá-lo, também, para o âmbito domiciliar. O processo de transição hospital para casa demanda apoio e oferta de cuidado centrado na família e na atenção domiciliar (AD)^(3)^. Esta constitui importante estratégia de atenção às CRIANES, por possibilitar a oferta de cuidados em seu ambiente social e familiar, e apoio longitudinal aos cuidadores. Apresenta, no Brasil, três modalidades: AD1, sob a responsabilidade da atenção primária; e AD2 e AD3, sob a responsabilidade do serviço de atenção domiciliar (SAD)^(6)^.

Estudo evidencia que a AD, além de promover desospitalizações, também centraliza o cuidado na família, oferecendo apoio ao desenvolvimento de habilidades com orientações necessárias para capacitar o cuidador principal, incluindo informações acerca dos sinais e sintomas de agudização da doença. Ademais, considera o ambiente domiciliar como elemento importante para a recuperação da criança, sendo capaz de proporcionar assistência ampliada, humanizada e integral por meio do estabelecimento do Projeto Terapêutico Singular (PTS)^(7)^, além de contribuir para a promoção do desenvolvimento infantil saudável.

No Brasil, a estruturação desse serviço é relativamente recente, de 2013, carecendo, portanto, de organização no trânsito e fluxo do hospital para o domicílio dos pacientes em geral e das CRIANES em particular. Estudo piloto desenvolvido no estado do Paraná, Brasil, elaborou uma proposta de fluxograma para atendimento de CRIANES no SAD^(8)^. Buscando ampliar o conhecimento sobre essa população em outros cenários nacionais, a validação do referido protocolo em outros estados, a exemplo da Paraíba, é uma estratégia que visa ampliar sua utilização.

Diante do exposto, surgiu a seguinte questão norteadora: o protocolo de fluxo de atenção às CRIANES é adequado para organizar o fluxo de atendimento nos SADs do estado da Paraíba?

## OBJETIVOS

Validar e implementar protocolo de fluxo de atenção às CRIANES.

## MÉTODOS

### Aspectos éticos

A pesquisa recebeu certificação do Comitê de Ética em Pesquisa. Foram respeitados todos os preceitos éticos da Resolução nº 466/2012. Para assegurar o anonimato, os participantes foram identificados com a letra inicial correspondente à categoria profissional (AS - assistente social; E -enfermeira; FI - fisioterapeuta; FO - fonoaudióloga; M - médica; N - nutricionista; P - psicóloga), seguido do número conforme a sequência de realização.

### Desenho, período e local do estudo

Trata-se de estudo de método misto sequencial explanatório QUAN→qual, desenvolvido em duas fases dependentes^(9)^. A pesquisa seguiu as recomendações do *checklist* para métodos mistos^(10)^. A etapa quantitativa foi necessária para a realização da validação de conteúdo do protocolo de fluxo para o SAD às CRIANES. A etapa qualitativa subsequente explorou as tendências do uso do protocolo de fluxo, bem como a interpretação das evidências dos dados quantitativos.

A coleta de dados ocorreu nos SADs habilitados, no estado da Paraíba, até julho de 2019, que atendiam à demanda espontânea de CRIANES, compondo um total de nove municípios. Entre esses municípios, 13 equipes incluíam as duas modalidades previstas: a equipe multiprofissional de atenção domiciliar (EMAD); e a equipe multiprofissional de apoio (EMAP).

### População ou amostra; critérios de inclusão e exclusão

Participou da etapa quantitativa como juiz um representante por equipe, totalizando 13 profissionais, que tinham titulação acadêmica em uma das categorias profissionais que compõem o SAD, com ou sem especialização, mestrado e/ou doutorado, e experiência profissional mínima de dois anos na assistência no SAD. Foram excluídos aqueles que estavam de licença e/ou férias durante o período da coleta de dados.

Na etapa qualitativa, foram realizadas oficinas de capacitação para os profissionais desenvolverem a implementação clínica do protocolo em SAD piloto do estado da Paraíba, Brasil, em março e abril de 2021. As oficinas ocorreram após agendamento prévio com 32 profissionais de nível superior que compunham a EMAD ou EMAP e que já haviam atendido ou estavam atendendo as CRIANES. Foram excluídos das oficinas os profissionais que não tinham disponibilidade de horário compatível com a pesquisadora e/ou que estavam de férias ou afastados do serviço.

### Protocolo do estudo

O estudo iniciou com o envio do convite de participação para os 13 profissionais dos SADs por correio eletrônico, bem como o instrumento padronizado para validação via *Google Forms^®^.* Nesse instrumento, o juiz informava suas características pessoais (sexo, idade), formação acadêmica (tempo de formação, tempo de atuação, especializações) e sua avaliação quanto aos aspectos referentes à pertinência, clareza, objetividade, simplicidade e precisão de 31 itens avaliados.

Após a análise da validação de conteúdo, foram realizados ajustes no item 3, no qual foram inseridos os termos “AD2” e “AD3”, no item 5, no qual foi incluída a possibilidade de encaminhamento por enfermeiros, médicos, fisioterapeutas, assistentes sociais, nutricionista e psicólogos do hospital e/ou APS, e no item 31, no qual foram inseridos os termos “AD2” e “AD3”. Em seguida, iniciou-se a etapa qualitativa.

Nessa etapa, foram realizadas seis oficinas com tempo médio de 60 minutos por encontro. Todas foram norteadas por um plano de intervenção previamente estruturado, baseado no referencial teórico da “aprendizagem significativa” de David Ausubel^(11)^. Dois meses após a implementação clínica do fluxograma, em outubro de 2021, os profissionais que haviam participado das oficinas e que haviam tido experiência no cuidado de CRIANES nos meses subsequentes às oficinas foram convidados a participar das entrevistas, compondo um total de 11 profissionais de saúde, atendendo ao critério de suficiência^(12)^.

As entrevistas foram realizadas pela pesquisadora principal, que tem experiência em pesquisa e que participou do SAD piloto, com agendamento prévio e em ambiente reservado. O roteiro utilizado foi dividido em dois blocos: o primeiro contém dados referentes às características sociodemográficas dos profissionais; e o segundo contém as seguintes questões norteadoras: como você avalia o funcionamento do protocolo de fluxo para o atendimento de CRIANES pelo SAD? Qual a sua percepção sobre a possibilidade de implantação do protocolo de fluxo para atendimento de CRIANES pelo SAD? Você modificaria algo no protocolo de fluxo para que ele melhor atendesse sua realidade? Se sim, o que e por quê?

Os áudios foram gravados em mídia digital e tiveram tempo médio de duração de 1 hora e 10 minutos. Após transcritas na íntegra, as entrevistas foram entregues aos participantes para que analisassem o conteúdo, podendo complementar ou refutar informações.

### Análise dos resultados e estatística

Na análise quantitativa, a concordância com os itens do instrumento foi avaliada pelo Índice de Validação de Conteúdo (IVC), com limite mínimo para a aceitação do item de 80%. Para a validação de construto do instrumento, utilizou-se a medida de confiabilidade das opiniões dos juízes, tendo sido realizada a confiabilidade expressa pela medida alfa de Cronbach, cujo valor foi de 0,87, demonstrando que o fluxograma possui boa fidedignidade segundo o julgamento dos juízes. Os cálculos foram realizados por meio da utilização do *software* estatístico R^(13)^ do *Statistical Package for the Social Sciences*, versão 20.0. Cada item avaliado será demonstrado no fluxograma por meio da Figura 1.

**Figura 1:** Fluxograma proposto para atendimento de crianças com necessidades de saúde especiais na atenção domiciliar, contendo 31 itens numerados e avaliados, João Pessoa, Paraíba, Brasil, 2022

*Fonte: Rossetto, Toso e Rodrigues (2020)^(8)^.*

Os dados qualitativos foram submetidos à análise temática indutiva, que se desenvolve em seis etapas^(14)^, tendo sido realizada inicialmente a familiarização com os dados das entrevistas, por meio da transcrição e leituras sucessivas com registro das primeiras impressões. Posteriormente, iniciaram-se a extratificação e codificação dos dados. Esses códigos foram agrupados de acordo com a afinidade para gerar pré-temas e temas. Ao final dessa fase, o material foi revisado para identificar lacunas no processo de estruturação dos recortes das entrevistas. Assim, foi possível identificar lacunas intratemas e intertemas para refiná-los, para que apresentassem um padrão coerente e coeso entre si e com os demais temas construídos. Em seguida, foram construídos os seguintes temas: *implementação do protocolo de fluxo de atenção às CRIANES: itens a serem aprimorados;* e *desafios para o SAD na gestão do cuidado às CRIANES segundo o protocolo de fluxo*. Ademais, todas as transcrições de áudio das entrevistas, bem como a avaliação dos temas, foram verificadas de forma consensual quanto à precisão por pelo menos duas autoras.

Após a análise dos dados quantitativos e qualitativos separadamente, foram realizadas metainferências a partir da mixagem e comparação dos valores do IVC, dos itens avaliados pelos juízes, e dos extratos de falas dos profissionais entrevistados. Os dados oriundos da análise dos itens convergentes e divergentes foram apresentados em *joint displays.*

## RESULTADOS

A integração dos dados quantitativos e qualitativos permitiu a identificação de pontos convergentes e divergentes entre a validação de conteúdo e análise da percepção dos profissionais. Com isso, identificou-se convergência dos dados no item 5 do protocolo, com IVC mediano e depoimentos que remetem à não utilização rotineira das fichas de encaminhamento das instituições ao SAD via e-mail. As fichas para acionamento do SAD são disponibilizadas via correio eletrônico ou entregues pessoalmente tanto pelo hospital quanto pela Atenção Primária à Saúde (APS), e algumas vezes, são realizados contatos telefônicos, havendo a necessidade de adequação do item.

No item 11, com relação ao preparo do cuidador, evidenciou-se a necessidade de complementação com a inclusão da análise do ambiente e estrutura do domicílio como condição importante para a alta hospitalar. Além disso, identificaram-se a preocupação em visitar o domicílio para avaliar as condições do ambiente para receber as CRIANES e a realização de pactuações com a rede de atenção diante das necessidades das crianças admitidas.

O item 14, que aborda a remoção hospitalar com veículo de transporte do SAD acompanhada por um profissional técnico em enfermagem, não foi validado em ambas as etapas da pesquisa. Evidenciou-se impossibilidade de implementá-lo por falta de transporte próprio do serviço devidamente equipado para essa finalidade e falta de segurança do profissional técnico em fazer a remoção.

O item 20, que orienta a comunicação mensal com a APS, foi avaliado como não adequado nas duas etapas da pesquisa. Os extratos qualitativos ampliam a possibilidade de interpretação ao evidenciar desarticulação entre o SAD e a APS, com comunicação não rotineira entre as equipes e desinteresse por parte da equipe da APS.

Por fim, no item 29, que orienta quanto à adaptação do cuidador como critério para a alta do SAD, também houve convergência em ambas as etapas, tendo em vista que esse foi considerado um critério suficiente para a alta segura. A apresentação dos dados supracitados consta no Quadro 1, no qual encontra-se o *joint display* com os dados convergentes.

**Quadro 1:** *Joint display* **de comparação de convergências entre o Índice de Validade de Conteúdo e narrativas nas entrevistas em profundidade com os profissionais do serviço de atenção domiciliar, João Pessoa, Paraíba, Brasil, 2021**

Itens	IVC	Narrativas nas entrevistas
Item 5 - SIM: Enfermeiros, médicos, fisioterapeutas, assistentes sociais, do hospital enviam ficha de encaminhamento por correio eletrônico para o SAD.	61,5	*Muitas vezes, não recebemos a ficha, recebemos mesmo a demanda. Eles vêm para agilizar o processo porque, muitas vezes, manda por correio eletrônico e demora a ser impresso. Então, eles ligam mesmo e já fazemos o agendamento da avaliação*. (E3)
Item 11 - Cuidador preparado? Executa todos os cuidados sem auxílio?	76,9	*Muitas vezes, são necessários aparelhos específicos para a criança ter condições de estar em casa. Analisamos essa real necessidade, para saber se têm condições de ser acompanhado pelo SAD no domicílio.* (N10) *A EMAD vai primeiro no hospital fazer o levantamento dos equipamentos e, se precisar de algum que não temos, acionamos os órgãos competentes para que realmente liberem os equipamentos e a criança possa ir para casa com mais segurança.* (P2)
Item 14 - Remoção com veículo de transporte de pacientes, do SAD ou do município, na data programada, acompanhada de técnico em enfermagem do SAD e com *kit* de materiais para o desenvolvimento dos cuidados domiciliares.	76,9	*Tem a questão do conselho de enfermagem* [...]. *Há um tempo, tivemos algumas dificuldades. Os técnicos de enfermagem daqui não se sentiam seguros se não estivessem com a enfermeira para estar removendo o paciente, porque os nossos pacientes são complexos, não são pacientes simples. A transferência de um paciente do ambiente hospitalar, que está estável, mas que a qualquer momento pode complicar.* (FI4) *Se o SAD não dispõe de transporte, nós não temos como fazer* [...]. *Do ponto de vista do fluxo* [do protocolo de fluxo], *é muito bom, mas precisamos adequar o ideal ao possível.* (M6)
Item 20 - Comunicação mensal com a unidade de atenção primária de referência sobre o acompanhamento da criança, por prontuário eletrônico ou correio eletrônico.	76,9	*Eu não faço mensal* [comunicação com a USF]. *Era bom que fosse, mas o “corre-corre” mesmo que não deixa. Tem muitos pacientes com muitas demandas, porque não são só crianças que atendemos.* (E9) *Não adianta ter essa iniciativa* [comunicação periódica]. *Já teve muito caso de, na prática, termos essa relação muito boa e buscarmos* [a USF] *e, de fato, não sermos bem recebidos. Se fosse passar, por exemplo, reuniões mensalmente, tenho certeza absoluta que eles nem olham, porque já levamos prontuário; as pessoas nem olham, nem para ver o papel.* (FI5)
Item 29: Cuidador adaptado aos cuidados?	92,3	*Se essa criança está estabilizada, a família bem orientada, segue os cuidados de forma adequada, sabemos que vai dar continuidade ao que foi orientado, e que tenha o apoio da unidade de saúde da família. Que esse serviço* [APS] *possa dar continuidade.* (N10)

*IVC - Índice de Validade de Conteúdo.*

No item 6, que aborda a avaliação de elegibilidade para admissão no SAD, foram identificadas divergências na comparação entre as etapas quantitativa e qualitativa, pois, apesar da validação dos juízes, a etapa qualitativa revelou que o tempo de até uma semana para a realização dessa avaliação não é cumprido.

Também foi identificada divergência no item 10, pois embora seja recomendado e tenha sido estabelecido no protocolo o preparo em conjunto entre hospital e equipe do SAD para a desospitalização da CRIANES, na prática assistencial, isso não aconteceu. Evidenciou-se a indisponibilidade para a preparação do cuidador em conjunto por profissionais da EMAD e do hospital, sendo os profissionais dos hospitais os responsáveis por esse preparo antes da alta.

Sobre a orientação de desenvolvimento do PTS pela EMAD juntamente com o cuidador, na primeira visita domiciliar (VD) após a admissão, como sugerido pelo item 16 do fluxograma, anteriormente validado pelos juízes, também foram identificadas divergências relacionadas à construção do PTS, ou seja, à periodicidade da realização, ao tempo de uma semana para a primeira discussão de PTS e à participação da família na construção.

O item 18 aborda a comunicação sobre a admissão à APS, via prontuário ou correio eletrônico com compartilhamento do PTS, validado na etapa quantitativa, mas divergente na etapa qualitativa por falta de comunicação regular com a APS, impossibilidade de utilização das vias eletrônicas de comunicação formal, e dificuldade para compartilhamento de responsabilidade entre o SAD e a APS.

Quanto à periodicidade da VD e aos cuidados ao cuidador, o item 29 foi validado pelos juízes, mas não implementado na prática assistencial. As divergências encontradas foram relacionadas à periodicidade da VD e ao suporte ao cuidador para atender suas demandas de saúde, pois falta articulação para garantir acesso aos serviços de saúde.

No item 23, relativo ao acionamento do Serviço de Atendimento Móvel de Urgência (SAMU), percebeu-se dificuldade ou mesmo recusa de atendimento para os casos de desestabilização da criança. O SAMU deveria ser o apoio do SAD em horários em que não há atendimento do serviço.

Foram identificados empecilhos para a realização do teleatendimento por atendente capacitado no item 25 do protocolo de fluxo. Os participantes alegaram carência de profissional exclusivo para essa atividade, indicando necessidade de contratação de profissionais para a devida implementação do teleatendimento. Essas divergências estão demonstradas no Quadro 2.

**Quadro 2:** *Joint display* **de comparação de divergências entre o Índice de Validade de Conteúdo e narrativas nas entrevistas em profundidade com os profissionais do serviço de atenção domiciliar, João Pessoa, Paraíba, Brasil, 2021**

Itens	IVC	Narrativas nas entrevistas
Item 6 - Realizar avaliação de elegibilidade, entre um e sete dias, por profissionais de nível superior da EMAD.	100	*A gente tenta fazer o mais rápido possível* [avaliação da elegibilidade]. *A gente não faz muito em até uma semana. Depende se a gente está com muito paciente, que já tenha uma agenda* [de atendimento anteriormente] *programada.* (M6)
Item 10 - Preparo do cuidador, pelos profissionais de nível superior da EMAD e do hospital, para os cuidados que serão necessários no domicílio. Orientação e demonstração dos cuidados.	100	*A equipe do hospital treina* [no ambiente hospitalar para a alta]. *A gente termina de treinar no domicílio, porque a gente não pode intervir na conduta dos profissionais do hospital. Então, a gente faz esse treinamento quando a gente está no domicílio* [com a criança]. *Mas quem faz o treinamento do cuidador, o paciente ainda estando hospitalizado, é a equipe do hospital.* (FI4)
Item 16 - Desenvolvimento do PTS pela EMAD, juntamente com o cuidador, na primeira VD após a admissão, em até uma semana, abordando diagnóstico, definição de metas, divisão de responsabilidades e tarefas e plano de reavaliação.	100	*Eu ainda não consegui fazer junto com o cuidador* [a construção do PTS]. *Essa proposta* [da primeira visita da em até uma semana a partir do acionamento] *não consegue ser alcançada. Se estender um pouquinho mais o tempo, conseguimos inserir duas profissionais para estarem fazendo essa visita, mas, em sete dias, não consegue. Duas semanas é o suficiente.* (E3)
Item 18 - Comunicar a unidade de atenção primária de referência sobre a admissão no SAD, via prontuário eletrônico ou correio eletrônico, com relato de caso e o PTS, requisitando contribuição da unidade na divisão de responsabilidades.	92,3	*Isso não é feito regularmente* [comunicação com a APS]. *Mas aquilo que a gente sente que precisa compartilhar* [a depender de necessidades específicas identificadas], *trata logo de agendar, como também no momento que precisa fechar a nossa permanência ali* [quando ocorre resolutividade da demanda]. *Então, geralmente, a gente faz o contato.* (M6)
Item 19 - Manter VD no mínimo semanal, observando a execução dos cuidados, a evolução da criança e orientando novos cuidados, se necessário. Identificar necessidade de suporte profissional para a família, providenciando-o na rede.	100	*Geralmente, é semanal* [periodicidade das visitas do SAD], *mas vai depender do quadro clínico.* (E7) *Não buscamos um profissional para o cuidador. Às vezes, oriento uma medicação; se houver necessidade, um antidepressivo. Já falo que ela precisa de um psicólogo, que temos na nossa equipe e pode dar um suporte a ela. Agora, se for um outro tipo de profissional, incentivamos ir ao médico do ESF* [Estratégia Saúde da Família] *para ele atender.* (M11)
Item 23 - SIM: Cuidador entra em contato com o SAMU, que fornecerá apoio também para os horários em que não há atendimento no SAD. Após atendimento de urgência/emergência no domicílio ou hospital, a criança retorna para atendimento pelo SAD.	100	*O paciente fala assim: “Ah, eu chamei o SAMU, mas o SAMU não veio, o SAMU disse que isso não era o serviço dele”. É como se o serviço de emergência sempre desacreditasse que aquele paciente realmente está precisando do serviço. A história de muitos telefonemas falsos criou uma barreira para que os profissionais vissem aquele chamado como algo realmente necessário.* (M6)
Item 25 - Atendente capacitado realiza orientações por telefone e, se necessário, é reavaliado pela EMAD ou EMAP.	84,6	*A pessoa que atende não é capacitada para orientar o cuidador. A família entra em contato com a recepção. A recepção atende, anota e passa a situação para nós* [profissionais de saúde], *e entramos em contato de acordo com a demanda.* (N10)

*Nota: IVC - Índice de Validade de Conteúdo.*

## DISCUSSÃO

A mixagem dos dados quantitativos e qualitativos possibilitou a compreensão dos ajustes necessários para adequar o protocolo de fluxo de atendimento às CRIANES à realidade prática, bem como identificar os óbices que interferem na sua funcionalidade.

Os primeiros elementos com necessidade de ajuste do protocolo avaliado dizem respeito ao acionamento e ao tempo para avaliação da elegibilidade das CRIANES. O programa “Melhor em Casa” permite e incentiva o acionamento do SAD pelos serviços de nível hospitalar, pela APS, pelas unidades de pronto atendimento, unidades de especialidades e de referências ambulatoriais, ou até mesmo por iniciativa e necessidade do paciente, familiares e vizinhos. Recomenda-se ainda que haja uma organização dos SADs para avaliação da elegibilidade, com prazos estabelecidos^(6)^.

O protocolo propõe um período de até sete dias para realização dessa avaliação. No entanto, a necessidade de adequações nas agendas das equipes, para atender às solicitações do hospital e APS, gerou a necessidade de adequação do tempo de avaliação de elegibilidade para até 15 dias após o acionamento do SAD.

Após a avaliação de elegibilidade, é necessário realizar a identificação de um cuidador responsável pelas CRIANES. O protocolo propõe que o preparo do cuidador seja efetuado em conjunto pela equipe do hospital e do SAD, modificando o modelo predominantemente biomédico da alta hospitalar, oportunizando a organização da alta junto à equipe multidisciplinar. Estudo internacional aponta as transições de cuidado hospital-domicílio-AD como possível causa de reinternações nas crianças com condições crônicas, sugerindo melhorias na comunicação como estratégia para a continuidade do cuidado^(15)^.

Na realidade brasileira, pesquisa constatou que a desospitalização de CRIANES pode ser influenciada pela ausência de um planejamento de acordo com a realidade da criança/família, bem como por altas não programadas que geram problemas na comunicação entre equipes, rede de saúde e família. Sugere ainda a implementação de protocolos institucionais como potencialidade para a melhoria das desospitalizações^(16)^. O preparo do cuidador para o desenvolvimento dos cuidados no domicílio, por profissionais do SAD e do hospital, contempla a interprofissionalidade para a continuidade do cuidado de forma segura, permitindo a programação de alta com organização do SAD para acompanhar a saída do hospital e efetuar o acolhimento no domicílio^(17)^. Uma estratégia para a efetivação deste item pode ser a utilização de videoconferências em dias anteriores à alta hospitalar; isso traria benefícios como o compartilhamento, a transparência e a humanização do cuidado^(18)^.

Outro elemento proposto no protocolo, relacionado à programação de alta de CRIANES, é a realização do transporte após a alta por ambulância do SAD ou transporte sanitário do município, acompanhado por um técnico de enfermagem. A análise da avaliação desse item permitiu identificar convergência na comparação entre validação de conteúdo e implementação clínica. Em ambas, foi evidenciada a inadequação do item para a realidade estudada, por inexistência de ambulância exclusiva para o SAD, por insegurança e por dúvidas acerca da regulamentação do Conselho Federal de Enfermagem (COFEN) para a realização desse tipo de transporte acompanhado por um profissional técnico de enfermagem.

O COFEN, na Resolução nº 588/2018, autoriza o profissional técnico de enfermagem a fazer o transporte de pacientes em cuidados mínimos ou intermediários e incumbe ao enfermeiro a avaliação do estado geral do paciente, a provisão de equipamentos necessários à assistência durante o transporte, a definição dos profissionais de enfermagem que assistirão o paciente durante o translado^(19)^ e a realização de educação permanente como estratégia para possibilitar a implementação do item^(20)^.

Com relação à aquisição de transporte equipado, a viabilidade demanda articulação da gestão do SAD e gestão municipal, com planejamento e organização financeira do município, a partir da inclusão da compra do veículo na Lei Orçamentária Anual seguida da efetivação de um processo licitatório. Além disso, é necessário prover recursos materiais e capacitar a equipe do SAD para sistematizar o uso do transporte para as transferências do hospital para o domicílio, e realizações de outras transferências eletivas sempre que necessário. Estudo de revisão de literatura comprovou a necessidade de reorganização dos recursos financeiros do Sistema Único de Saúde (SUS), devido ao desfinanciamento de políticas públicas, que têm prejudicado o direito à saúde e à atenção integral^(21)^. Destaca-se a importância da reorganização orçamentária, com a finalidade de obter veículos específicos para o SAD que viabilizem transporte seguro de pacientes acompanhados pela atenção básica, tendo em vista que os serviços de transporte sanitário e o serviço móvel de urgência já se responsabilizam pelo suporte ao serviço terciário e ambulatorial em remoções para realização de exames e outros procedimentos, bem como transferências.

Além da remoção nas altas, as CRIANES atendidas pelo SAD também necessitam, eventualmente, de transporte para realização de exames e/ou terapias complementares. A portaria ministerial que redefine a AD orienta, como prioridade dos gestores municipais de saúde, o fluxo de transporte e a remoção do usuário tanto em situações eletivas quanto em situações de urgência^(22)^. Ademais, a Portaria MS nº 2.563/2017 garante o financiamento para a utilização de transporte sanitário eletivo destinado aos usuários do SUS^(23)^. A realidade do funcionamento é permeada, contudo, por sérias dificuldades relacionadas à utilização de transporte para desospitalização e remoção de CRIANES, tanto nas situações eletivas quanto nas situações de urgência.

Identificaram-se também lacunas na comunicação entre os serviços da Rede de Atenção à Saúde (RAS), pois nem as unidades de saúde, nem os SADs possuem prontuário eletrônico implantado, o que prejudica, consideravelmente, a comunicação em rede. Entretanto, essa realidade está em processo de mudança, devido ao início da implementação da Política de Saúde Digital pelo Ministério da Saúde (MS), que tem como prioridade a informatização dos três níveis de atenção até 2028^(24)^, tendo em vista que a capacidade comunicativa proporcionada pela utilização do prontuário eletrônico facilita a comunicação e a troca de informações entre as equipes de saúde^(25)^.

Além disso, estudo brasileiro identificou como desafios para a comunicação^(26)^ a falta de qualificação dos profissionais da APS para a coordenação do cuidado, a falta de compreensão acerca dos critérios de elegibilidade pelo SAD associada à ausência de direcionamento para a manutenção da comunicação entre os pontos da RAS, bem como a desproporcionalidade entre a capacidade de atendimento e a população que necessita ser assistida.

Pesquisa realizada com profissionais da APS destaca como estratégia para superar esse entrave na comunicação a organização de espaços para reuniões formais entre as equipes, bem como a necessidade de políticas públicas que garantam mecanismos para o trabalho colaborativo na APS^(27)^. A comunicação é elemento estratégico e essencial para a atenção integral da equipe multidisciplinar, mas dependente de vínculo profissional e espaço de comunicação entre profissionais e gestores^(28)^.

Nessa perspectiva, o meio digital é um espaço que pode ser utilizado para melhorar a comunicação entre os profissionais da APS e do SAD por meio da criação de uma plataforma de comunicação formal através de aplicativos de mensagens, no qual haveria a troca de informações sobre as necessidades de saúde do paciente, facilitando a continuidade do cuidado e a comunicação entre as equipes. Para isso, apesar dos atuais investimentos do MS em tecnologias da informação e comunicação, ainda são necessárias estratégias governamentais e políticas com vistas a ampliar o acesso, a capacitação e a articulação entre os profissionais de saúde em seus respectivos serviços públicos de atuação^(29)^.

No tocante ao desenvolvimento do PTS na primeira visita, foi possível identificar a ausência de realização de modo sistematizado e rotineiro, além de não ser considerado para a comunicação com a APS. A utilização dessa ferramenta norteia a abordagem da complexidade do cuidado, considerando as questões clínicas, familiares, espirituais e sociais que estão envolvidas nas condições que alteram a saúde, permitindo também o levantamento das demandas das CRIANES e das famílias, direcionando-os na RAS, além de orientar a organização e a periodicidade das VDs, facilitando a troca de saberes, tomada de decisão e singularidade da atenção prestada^(26)^.

Quanto à periodicidade das visitas, foi identificada variabilidade na rotina, a depender da avaliação do profissional de saúde acerca da necessidade das CRIANES. Estudo realizado com os familiares de CRIANES atendidas pelo SAD apontou que a flexibilização dos atendimentos semanais é algo benéfico, pois possibilita a continuidade do cuidado domiciliar ao se adequar à rotina familiar^(30)^.

Cabe destacar que o protocolo não propõe o enrijecimento das ações de cuidado, sendo compreensível a adequação da quantidade máxima de visitas que podem ser realizadas pelas equipes para atender às demandas da CRIANES. Contudo, é necessário padronizar o período mínimo para realização das visitas de, pelo menos, uma visita por semana, além de transferir os pacientes com necessidade menor que quatro visitas por mês para a AD1^(6)^.

Nessa perspectiva, há necessidade de adequação do processo de trabalho das equipes para atendimento da meta mínima proposta pelo MS. O protocolo proposto constitui-se como ferramenta facilitadora nesse processo, com avaliação frequente da realidade situacional, transferindo para a APS as CRIANES que não necessitam mais de atendimento nas modalidades AD2 e AD3. O instrumento proposto pode ser flexibilizado a partir da análise das exceções e das especificidades de cuidado, tendo como base intervencional o PTS construído de modo compartilhado entre as equipes e a família.

Outro ponto divergente se relaciona às situações em que ocorre agudização da doença nas CRIANES acompanhadas pelo SAD. Apesar de a portaria ministerial garantir a provisão de transporte e acesso aos serviços de urgência, observaram-se empecilhos importantes no acionamento do SAMU, com falta de credibilidade dos familiares quando precisam de ambulância.

Vale ressaltar que os profissionais do SAMU também enfrentam dificuldades, em seu cotidiano de trabalho, associadas ao desconhecimento da população em relação à função do SAMU, às lacunas no processo de regulação e, algumas vezes, à inadequação dos recursos disponíveis com má distribuição das ambulâncias. A resolução dessa problemática demanda estratégias que melhorem a comunicação entre os profissionais do SAMU e demais profissionais da RAS^(31)^.

A disponibilização de teleatendimento pelo SAD pode se constituir como estratégia que pode influenciar diretamente o acionamento adequado ou inadequado do SAMU. O teleatendimento é abordado no protocolo de fluxo como estratégia para esclarecimento de dúvidas, sem caráter de urgência ou emergência^(8)^. Entretanto, foram identificados óbices para a efetivação do teleatendimento por falta de atendente capacitado disponível para o desenvolvimento dessa função e pela ausência de telefone móvel para tal finalidade. Estudo de revisão sistemática demonstra a utilização da estratégia de suporte remoto aos pacientes com condições crônicas associada a uma melhor qualidade de vida^(32)^.

A respeito da alta das CRIANES do SAD, identificou-se que a adaptação do cuidador para continuidade dos cuidados no domicílio foi considerada um critério válido e suficiente para o encaminhamento das CRIANES para a AD1. Entretanto, a falta de confiança e a ausência de vínculo entre CRIANES, suas famílias e a APS são identificadas como condições que causam a transferência da responsabilidade da APS para os serviços de atenção secundária, por despreparo e insegurança dos profissionais de saúde e por desarticulações da RAS, prejudicando a longitudinalidade do cuidado^(33)^.

Refletir a implementação clínica do protocolo de fluxo proposto aponta dificuldades do cotidiano da prática. Entretanto, sua utilização tem como finalidade garantir direitos, de modo igualitário, dentro da RAS a toda a população de CRIANES assistidas.

### Limitações do estudo

Reconhece-se como limitações deste estudo a realização da implementação em apenas um município do estado, decorrente das dificuldades enfrentadas para a realização da pesquisa em período pandêmico (COVID-19). Destaca-se a importância da avaliação do fluxograma em outros municípios e estados para uma possível adequação para a utilização em território nacional com as devidas articulações e pactuações com os demais pontos da RAS.

### Contribuições para as áreas da enfermagem, saúde ou políticas públicas

A validação do protocolo de fluxo para atendimentos de CRIANES auxiliou a compreender a complexidade do cuidado de CRIANES assistidas pelo SAD. O protocolo organizativo potencializará os recursos em saúde, com empoderamento de profissionais e usuários, além de fornecer subsídios para o gerenciamento do fluxo de CRIANES no SAD, contribuindo para a longitudinalidade do cuidado ofertado a essa população.

## CONCLUSÕES

A adequação do protocolo de fluxo proposto está condicionada a ajustes organizacionais, a exemplo da inserção do cuidador no levantamento das necessidades das CRIANES, ampliação da quantidade de dias para avaliação da elegibilidade, e adequação das agendas para periodicidade das visitas e reuniões de equipe. Ademais, são necessários ajustes na articulação entre SAD, hospital e APS, bem como o provimento de materiais e recursos para o funcionamento adequado do SAD.

Os ajustes no protocolo de fluxo para garantir a funcionalidade da tecnologia em saúde proposta são uma demanda complexa, mas factível e útil para o cenário estudado. No entanto, cabe destacar que as barreiras estruturais e comunicacionais identificadas geram sobrecarga de equipamentos e serviços de saúde, bem como comprometem a continuidade do cuidado de CRIANES entre os diferentes pontos da RAS. Após a análise comparativa dos dados e o entendimento dos fatores que influenciam a aplicabilidade prática imediata, o protocolo de fluxo de atenção às CRIANES foi avaliado como funcional para utilização pelo SAD no cuidado dessas crianças. Esse cuidado é um processo dinâmico e interativo que requer articulação, empatia e gestão pública de qualidade.

## Data Availability

Os dados da pesquisa estão disponíveis no corpo do artigo.
